# Impact of center volume on in-hospital mortality in adult patients with out‑of‑hospital cardiac arrest resuscitated using extracorporeal cardiopulmonary resuscitation: a secondary analysis of the SAVE-J II study

**DOI:** 10.1038/s41598-024-58808-y

**Published:** 2024-04-09

**Authors:** Kayo Misumi, Yoshihiro Hagiwara, Takuya Kimura, Toru Hifumi, Akihiko Inoue, Tetsuya Sakamoto, Yasuhiro Kuroda, Takayuki Ogura, the SAVE-J II. study group, Hirotaka Sawano, Yuko Egawa, Shunichi Kato, Kazuhiro Sugiyama, Naofumi Bunya, Takehiko Kasai, Shinichi Ijuin, Shinichi Nakayama, Jun Kanda, Seiya Kanou, Toru Takiguchi, Shoji Yokobori, Hiroaki Takada, Kazushige Inoue, Ichiro Takeuchi, Hiroshi Honzawa, Makoto Kobayashi, Tomohiro Hamagami, Wataru Takayama, Yasuhiro Otomo, Kunihiko Maekawa, Takafumi Shimizu, Satoshi Nara, Michitaka Nasu, Kuniko Takahashi, Reo Fukuda, Shinichiro Shiraishi, Ryosuke Zushi, Norio Otani, Migaku Kikuchi, Kazuhiro Watanabe, Takuo Nakagami, Tomohisa Shoko, Nobuya Kitamura, Takayuki Otani, Yoshinori Matsuoka, Masaaki Sakuraya, Hideki Arimoto, Koichiro Homma, Hiromichi Naito, Shunichiro Nakao, Tomoya Okazaki, Yoshio Tahara, Hiroshi Okamoto, Jun Kunikata, Hideto Yokoi

**Affiliations:** 1 Department of Emergency and Critical Care, Saiseikai Utsunomiya Hospital, 911-1, Takebayashi-machi, Utsunomiya, Tochigi 321-0974 Japan; 2 Department of Cardiology, Saiseikai Utsunomiya Hospital, Utsunomiya, Japan; 3 Department of Emergency and Critical Care Medicine, St. Luke’s International Hospital, Tokyo, Japan; 4 Department of Emergency and Critical Care Medicine, Hyogo Emergency Medical Center, Kobe, Japan; 5 Department of Emergency Medicine, Teikyo University School of Medicine, Tokyo, Japan; 6 Department of Emergency Medicine, Kagawa University School of Medicine, Kagawa, Japan; 7 Osaka Saiseikai Senri Hospital, Suita, Japan; 8 Saitama Red Cross Hospital, Saitama, Japan; 9 Tokyo Metropolitan Bokutoh Hospital, Tokyo, Japan; 10 Sapporo Medical University, Sapporo,, Japan; 11 Hyogo Emergency Medical Center, Kobe, Japan; 12 Teikyo University Hospital, Tokyo, Japan; 13 Nippon Medical School, Tokyo, Japan; 14 National Hospital Organization Disaster Medical Center, Tachikawa, Japan; 15 Yokohama City University Medical Center, Yokohama, Japan; 16 Toyooka Public Hospital, Toyooka, Japan; 17 Tokyo Medical and Dental University Hospital of Medicine, Tokyo, Japan; 18 Hokkaido University Hospital, Sapporo, Japan; 19 Teine Keijinkai Hospital, Sapporo, Japan; 20 Urasoe General Hospital, Urasoe, Japan; 21 Nippon Medical School Tama Nagayama Hospital, Tama, Japan; 22 Aizu Central Hospital, Fukushima, Japan; 23 Osaka Mishima Emergency Critical Care Center, Takatsuki, Japan; 24 St. Luke’s International Hospital, Tokyo, Japan; 25 Dokkyo Medical University, Mibu, Japan; 26 Nihon University Hospital, Tokyo, Japan; 27 Omihachiman Community Medical Center, Omihachiman, Japan; 28 Tokyo Women’s Medical University Medical Center East, Tokyo, Japan; 29 Kimitsu Chuo Hospital, Kisarazu, Japan; 30 Hiroshima City Hiroshima Citizens Hospital, Hiroshima, Japan; 31 Kobe City Medical Center General Hospital, Kobe, Japan; 32 JA Hiroshima General Hospital Hiroshima, Hatsukaichi, Japan; 33 Osaka City General Hospital, Osaka, Japan; 34 Keio University School of Medicine, Tokyo, Japan; 35 Okayama University Hospital, Okayama, Japan; 36 Osaka University Graduate School of Medicine, Suita, Japan; 37 Kagawa University Hospital, Miki, Japan; 38 National Cerebral and Cardiovascular Center, Suita, Japan

**Keywords:** Post-cardiac arrest syndrome, Cardiopulmonary arrest, ECMO, ECMO center, High volume center, Cardiology, Cardiac device therapy

## Abstract

Recently, patients with out-of-hospital cardiac arrest (OHCA) refractory to conventional resuscitation have started undergoing extracorporeal cardiopulmonary resuscitation (ECPR). However, the mortality rate of these patients remains high. This study aimed to clarify whether a center ECPR volume was associated with the survival rates of adult patients with OHCA resuscitated using ECPR. This was a secondary analysis of a retrospective multicenter registry study, the SAVE-J II study, involving 36 participating institutions in Japan. Centers were divided into three groups according to the tertiles of the annual average number of patients undergoing ECPR: high-volume (≥ 21 sessions per year), medium-volume (11–20 sessions per year), or low-volume (< 11 sessions per year). The primary outcome was survival rate at the time of discharge. Patient characteristics and outcomes were compared among the three groups. Moreover, a multivariable-adjusted logistic regression model was applied to study the impact of center ECPR volume. A total of 1740 patients were included in this study. The center ECPR volume was strongly associated with survival rate at the time of discharge; furthermore, survival rate was best in high-volume compared with medium- and low-volume centers (33.4%, 24.1%, and 26.8%, respectively; *P* = 0.001). After adjusting for patient characteristics, undergoing ECPR at high-volume centers was associated with an increased likelihood of survival compared to middle- (adjusted odds ratio 0.657; *P* = 0.003) and low-volume centers (adjusted odds ratio 0.983; *P* = 0.006). The annual number of ECPR sessions was associated with favorable survival rates and lower complication rates of the ECPR procedure.

*Clinical trial registration*: https://center6.umin.ac.jp/cgi-open-bin/ctr_e/ctr_view.cgi?recptno=R000041577 (unique identifier: UMIN000036490).

## Introduction

The number of patients experiencing out-of-hospital cardiac arrest (OHCA) is increasing^1–3^. Patients with OHCA who are refractory to conventional resuscitation have recently started undergoing extracorporeal cardiopulmonary resuscitation (ECPR), with previous studies showing that ECPR decreases in-hospital mortality compared with conventional resuscitation^4,5^.

Management of mechanical devices, including extracorporeal membrane oxygenation (ECMO), has a high complication rate^6,7^ and requires specialized knowledge and skills for effective and safe management. However, despite the difficulties of ECPR and post-resuscitation management with ECMO, there are no widely used guidelines for ECPR and ECMO management. Therefore, physicians administering ECPR must be proficient in ECMO circuit management and post-resuscitation care, and well trained in veno-arterial cannulation to prevent complications. Considerable experience and learning curves are required to improve ECPR management skills and may be related to the annual ECPR cases number performed at each hospital. Nonetheless, the association between annual center volume with in-hospital mortality in adult patients with OHCA resuscitated using ECPR has not been clarified. Therefore, this study aimed to investigate the effect of center ECPR volume on outcomes in patients with OHCA who underwent ECPR.

## Materials and methods

### Study design and cohort

To examine whether center ECPR volume was associated with survival rate at the time of discharge in patients with OHCA who underwent ECPR, we used a dataset from the SAVE-J II study cohort. The study design has been described in detail elsewhere^8,9^. In brief, this cohort derived from a retrospective multicenter study in Japan that included 36 university and community hospitals and enrolled 2,157 consecutive patients with OHCA aged ≥ 18 years who were resuscitated using ECPR between January 1, 2013, and December 31, 2018. In the SAVE-J II study, ECPR was defined as resuscitation with veno-arterial ECMO in patients with refractory cardiac arrest. Due to the retrospective design of this study, the implementation of ECPR lacked specific criteria and was depended on the judgement of each institution.

This study named Study of Advanced Cardiac Life Support for Ventricular Fibrillation with Extracorporeal Circulation in Japan was retrospectively approved by the Institutional Ethics Committee of Kagawa University (approval number: 2018-110, approved date: 15 April 2019) and that of each participating institution. This secondary analysis of de-identified data was approved by the Institutional Review Board of the Saiseikai Utsunomiya Hospital (approval number: 2023-06). The requirement for informed consent was waived by the ethics committee due to the retrospective nature of the study. This study was performed in accordance with the 1975 Declaration of Helsinki Guidelines for Clinical Research Protocols.

### Study population

From all the patients in the SAVE-J II registry, the present study excluded those who did not meet the ECPR criteria, such as those who withdrew after cannulation and before turning the ECMO pump on due to the return of spontaneous circulation (ROSC), those who received ECPR after intensive care unit admission, those who achieved ROSC before cannulation, and those who were transferred to the participating institutions from another hospital, as it precluded the definition of center volume. We divided the remaining patients into three groups according to the tertile of center ECPR volume. As a result, three groups were defined as follows: (1) high-volume centers (HVC) (≥ 21 ECPR sessions/year), (2) medium-volume centers (MVC) (11–20 sessions/year), and (3) low-volume centers (LVC) (< 11 sessions/year). For adequate background comparison, we further excluded patients with non-cardiac conditions, including acute aortic syndrome/aortic aneurysm, hypothermia, primary cerebral disorder, infection, drug intoxication, trauma, suffocation, drowning, and other external causes, and patients with missing outcome data. Missing data were not replaced or estimated.

### Outcome measurements

The primary endpoint of this study was survival rate at the time of discharge, and the secondary endpoints were the proportion of complications during ECPR cannulation and ECMO management and favorable neurological outcomes at the time of discharge. The complications of ECPR cannulation included malpositioning of the cannula, unsuccessful cannulation, and cannulation-related bleeding. Cannula malposition was defined as cannulation requiring correct positioning or cannulation of the wrong vessel, such as arterial-arterial or veno-veno cannulation. Unsuccessful cannulation was defined as a failure to complete cannulation. Cannulation-related bleeding included cannulation site bleeding and retroperitoneal hemorrhage requiring blood transfusion or surgical intervention/interventional radiology, and other forms of hemorrhage included intracerebral hemorrhage confirmed on computed tomography (CT), mediastinal hemorrhage, intra-abdominal organ hemorrhage, and gastrointestinal hemorrhage requiring blood transfusion or surgical and radiological intervention. Complications during ECMO management included hemorrhage, ischemia, and ECMO equipment problems. The Cerebral Performance Categories (CPC) scale was used to classify the neurological outcomes as follows: CPC 1, full recovery; CPC 2, moderate disability; CPC 3, severe disability; CPC 4, coma or vegetative state; and CPC 5, death. CPCs 1–2 were considered favorable outcomes, and CPCs 3–5 represented unfavorable outcomes^10^.

### Statistical analysis

Continuous variables were expressed as medians with interquartile ranges. Categorical variables were expressed as numbers and percentages. Patient characteristics and outcomes were evaluated among the three groups using the Kruskal–Wallis test for continuous variables and the chi-square or Fisher’s exact test for dichotomous variables. Multivariate analysis to identify predictors of in-hospital mortality was performed using linear regression of clinically important variables clustered by ECPR center volume, such as age, gender, incidence of witnessed cardiac arrest, bystander cardiopulmonary resuscitation (CPR), and initial cardiac rhythm at the scene. Moreover, a multivariable-adjusted logistic regression model was applied to study the impact of center ECPR volume by using age, gender, incidence of witnessed cardiac arrest, bystander cardiopulmonary resuscitation (CPR), initial cardiac rhythm and as confound factors. Regression models were allied with adjusted odds ratios (AOR) and with 95% confidence intervals (CI). Differences were considered statistically significant for two-tailed *P*-values < 0.05. All analyses were performed using the R software (version 4.2.1; R Foundation for Statistical Computing, Vienna, Austria).

## Results

**Figure 1:**
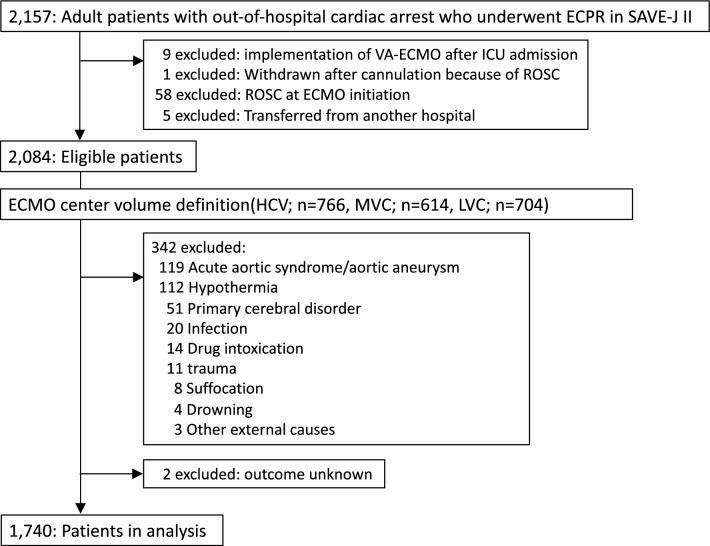
Flowchart of study participants enrollment. *ECPR* extracorporeal cardiopulmonary resuscitation, *ECMO* extracorporeal membrane oxygenation, *VA-ECMO* veno-arterial ECMO, *ICU* intensive care unit, *ROSC* return of spontaneous circulation, *HVC* high-volume center, *MVC* medium-volume center, *LVC* low-volume center.

**Figure 2:**
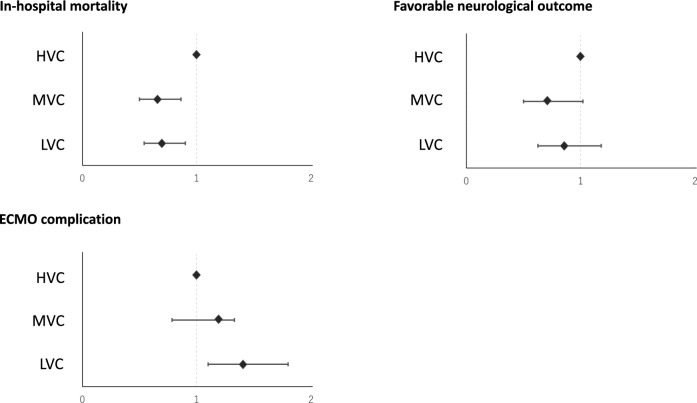
Adjusted odds ratios for logistic models of outcomes and various complications. The dashed lines indicate the high-volume centers used as reference. *HVC* high-volume center, *MVC* medium-volume center, *LVC* low-volume center.

Of the 2157 adult patients with OHCA in the SAVE-J II cohort, 2,084 were finally enrolled to assess the influence of center ECMO volume. The patients were divided into three groups according to the tertiles of center ECPR volume: 766 (36.8%) patients from 5 HVCs, 614 (29.5%) from 7 MVCs, and 704 (33.8%) from 24 LVCs (see Additional Fig. 1 in Additional file 1). After excluding patietns with cardiopulmonary arrest caused by non-cardiac conditions or external causes and missing outcome data, 1740 patients were finally analyzed (Fig. 1). The comparison of the baseline patient characteristics between the three ECPR center volumes is shown in Table 1. On average, the median age of the patients was 60 years old, and the proportion of male patients was 83.9%. Although the groups showed no significant difference in the time from onset to hospital arrival, the time from hospital arrival to ECMO was shorter in the HVC group. Outcomes are shown in Table 2. The center ECPR volume was strongly associated with survival rate at the time of discharge and was the higher in HVC compared with those at MVC and LVC: 33.4%, 24.1%, and 26.8%, respectively (*P* = 0.001). The proportion of total ECMO complications was significantly lower in HVC (27.8%) than in MVC (37.6%) and LVC (34.8%; *P* = 0.010). Moreover, multivariate analysis was used to account for variations in the background characteristics of the patients and the center ECPR volume (Table 3). After adjusting for the patient characteristics, undergoing ECPR at HVCs was associated with increased likelihood of survival compared with MVC (AOR 0.657; 95% CI 0.500–0.863, *P* = 0.003) and LVC (AOR 0.983; 95% CI 0.541–0.901, *P* = 0.006). Furthermore, it was also significantly associated with ECMO complications compared with LVCs (AOR, 1.410; 95% CI 1.100–1.800, *P* = 0.006), while not being associated with neurological outcomes (Fig. 2).

**Table 1:** Comparisons of baseline patient characteristics between groups.

Variables	HVC (n = 629)	MVC (n = 510)	LVC (n = 601)	*P* value
Average annual volume	26 (25, 27)	13 (13, 16)	7 (4,8)	< 0.001
Age, years	62 (50, 69)	61 (51, 69)	59 (47, 67)	< 0.001
Sex, male	523 (83%)	434 (85%)	356 (84%)	0.662
Witnessed cardiac arrest	525 (84%)	384 (76%)	457 (77%)	0.001
Bystander CPR	651 (57%)	129 (69%)	522 (56%)	0.129
Initial cardiac rhythm				
Shockable rhythm	435 (70%)	325 (65%)	620 (71%)	0.061
Pulseless electrical activity	152 (24%)	127 (26%)	120 (20%)	
Asystole	39 (6%)	45 (9%)	54 (9%)	
Cause of cardiac arrest				
Cardiac	540 (86%)	419 (83%)	535 (89%)	< 0.001
Pulmonary embolism	31 (5%)	16 (3%)	26 (4%)	
Non-cardiac cause	20 (3%)	12 (2%)	20 (3%)	
Unknown	38 (6%)	62 (12%)	20 (3%)	
Emergency coronary angiography	517 (82%)	336 (66%)	503 (84%)	< 0.001
Percutaneous coronary intervention	289 (46%)	205 (45%)	305 (51%)	0.083
Time course, minutes				
Time from onset until hospital arrival	33 (26, 42)	36 (27, 43)	33 (26, 42)	0.083
Time from onset until ECMO	51 (42, 61)	61 (51, 74)	66 (55, 82)	< 0.001
Time from hospital arrival until ECMO	16 (12, 22)	25 (19, 33)	30 (22, 44)	< 0.001
Estimated low-flow time	49 (41, 58)	58 (48, 69)	61 (51, 75)	< 0.001

Data are presented as the median [interquartile range], or n (%). *HVC* high-volume center, *MVC* medium-volume center, *LVC* low-volume center, *CPR* Cardiopulmonary resuscitation, *ECMO* extracorporeal membrane oxygenation.

**Table 2:** Outcome data and complications during extracorporeal cardiopulmonary resuscitation among each group.

Variables	HVC (n = 629)	MVC (n = 510)	LVC (n = 601)	*P* value
ECPR or ECMO management complications				
Overall^a^	175 (27.8%)	141 (37.6%)	209 (34. 8%)	0.010
Procedure related complications				
Cannula malposition	10 (1.6%)	44 (8.6%)	30 (5.0%)	< 0.001
Unsuccessful cannulation	3 (0.5%)	6 (1.2%)	4 (0.7%)	0.381
Cannulation related bleeding	89 (14.2%)	72 (14.2%)	92 (15.4%)	0.794
Others	23 (3.7%)	26 (5.1%)	24 (4.0%)	0.469
ECMO management related complications				
Hemorrhage	44 (7.0%)	26 (5.1%)	76 (12.8%)	< 0.001
Ischemia	14 (2.2%)	7 (1.4%)	10 (1.7%)	0.536
ECMO equipment trouble	4 (0.6%)	3 (0.6%)	22 (3.8%)	< 0.001
Others	11 (1.8%)	7 (1.4%)	5 (0.9%)	0.393
Outcomes				
Survival to hospital discharge, n (%)	210 (33.4%)	123 (24.1%)	161 (26.8%)	0.001
Favorable neurological outcome, n (%)	105 (16.7%)	62 (12.2%)	95 (15.8%)	0.085
Length of ECMO, days	4 (3, 5)	3 (2, 5)	3 (2, 5)	0.260
Length of hospital stay, days	5 (2, 25)	2 (1, 12)	4 (2, 23)	< 0.001
Length of hospital stay among survivors, days	32 (22, 53)	30 (26, 44)	40 (28, 60)	< 0.001

Data are presented as the median [interquartile range], or n (%). *HVC* high-volume center, *MVC* medium-volume center, *LVC* low-volume center, *ECMO* extracorporeal membrane oxygenation.

^a^Percentage of patients with complications (even if two or more complications, count as one).

**Table 3:** Center volume and adjusted survival outcome at the time of dischargement.

	OR	95% CI	*P* value
Center volume			
High-volume center	Reference		
Medium-volume center	0.657	0.500–0.863	0.003
Low-volume center	0.698	0.541–0.901	0.006
Age	0.983	0.975–0.991	< 0.001
Sex			
Male	Reference		
Female	1.45	1.080–1.940	0.013
Witness	1.43	1.070–1.920	0.016
By-stander CPR	1.2	0.955–1.510	0.118
Initial rhythm			
Shockable rhythm	Reference		
Pulseless electrical activity	0.339	0.204–0.562	< 0.001
Asystole	0.471	0.354–0.627	< 0.001

*CPR* cardiopulmonary resuscitation.

## Discussion

This study investigated the association between the center volume, survival rate, and other outcomes in patients with OHCA who underwent ECPR. Hospitals with the highest annual volume of ECPR had significantly higher survival rates at discharge and lower complication rates during ECPR and ECMO management.

The association between mortality and center ECPR volume has been previously evaluated in limited cohort. Tonna et al. indicated that the hospital-level ECPR annual case volume was associated with in-hospital survival^11^. However, that study only showed that center ECPR volume was one of the factors associated with in-hospital mortality, and detailed information related to the difference in the annual number of patients receiving ECPR was not described. Moreover, although Watanabe et al. mentioned the center ECMO volume in their study, they did not examine the association between center ECMO volume and outcomes^12^. Therefore, our study is the first to investigate the relationship between patient background, prognosis, multiple complications, detailed multiple complication and ECPR center volume based on the annual number of ECPR cases. In this study, we used tertiles to define the ECPR central volume, as there is no standard for defining the ECPR central volume. However, the number of each ECMO center volume was not so different from these previous studies^11,12^.

On the other hand, this study had a higher proportion of the patients with non-shockable rhythm than other studies. Although some typical patients groups who can gain the better clinical outcome from ECPR are well known and previous studies had chosen these patients in the prospective studies^5,13^, in the real clinical practice, ECPR is widely performed by the physicians judgments such as the patients that initial waveform was pulseless electrical activity(PEA) due to the pulmonary embolism or in patients without the shockable rhythm who are judged as treatable due to hypothermia^14,15^. In fact, the main article of SAVE-J II study showed that 9.2% of the patients with initial waveform PEA and 3.9% of patients with asystole had favorable neurological outcomes^8^. Therefore, this study was a retrospective observational study, and I guess this is a real world data.

The results of this study have several possible explanations. First, the HVC group had a significantly shorter time from hospital arrival to ECMO initiation. Based on this, it is expected that a HVC may decide to start ECPR earlier or that the time from ECPR start to pumping is shorter due to a more extensive experiences with ECPR. Second, although some studies have recently shown that ECPR decreased mortality in patients with OHCA who are refractory to conventional resuscitation the mortality rate in patients with OHCA who have undergone ECPR remains high^16,17^. This is not only due to the severity of the patient’s background but also the high complication rates during ECPR and ECMO management^6,18^. A previous meta-analysis has reported that mechanical cardiac devices tend to cause severe or life-threatening bleeding and peripheral vascular complications^6,19^. It also showed that fewer complications lead to decreased mortality in patients with veno-arterial ECMO^20^. Therefore, appropriate management of mechanical devices is required to both reduce avoidable complications and deal with them adequately if they occur. Furthermore, in other complex or specialized procedures, such as cardiovascular surgery or organ transplantation, patient outcomes have been reported to be largely dependent on center volume^21–23^. Therefore, institutions with a high annual number of ECPR cases can increase and maintain their management skills, which may lead to higher survival and lower complication rates. Here, it is also necessary to mention the impact of hospital size on this result or the proportion of the ECPR from all cardiac arrest patients at each hospital. However, as this study only enrolled patients who underwent ECPR, it is impossible to show a relationship between hospital capacities and ECPR center volume. Therefore, it remains unclear whether the hospital with higher number of ECPR annual cases lead to better clinical outcomes due to the availability of hospital resources for treatment, or whether even relatively small hospitals with a high number of ECPR cases can achieve sufficient outcomes. As such, further studies in this point are warranted.

However, if patients with OHCA are consolidated into an HVC, it might prolong the time from onset to ECMO and reduce flow time. The combination of expeditious coronary angiography and admission to an invasive heart center has been demonstrated to improve survival in patients with OHCA^24^, and another study showed that hospital survival is more strongly associated with post-resuscitation care than with acute resuscitation skills in the emergency room^25^. Accordingly, transportation to high-volume ECMO centers after the prompt introduction of ECPR at prehospital care or regional hospitals may lead to improved survival rates. Future studies are needed to examine how to balance expeditious arrest-to-cannulation times and the consolidation of ECPR.

### Limitations

This study has some limitations. First, as this was a retrospective observational study, the inclusion criteria were not defined and the indications for performing ECPR, including patient selection and timing of ECMO insertion, varied by physiatrist and institution. Therefore, selection bias is inevitable and there might be some variation in the selection of patients undergoing ECPR between institutions. However, the survival rate of the patients undergoing ECPR in this study did not differ much compared to previous retrospective and prospective ECPR registries^5,26^. Nevertheless, this point should be acknowledged. Second, we used a Japanese cohort, and the Japanese emergency medical system and ECPR methods were different from those of American and European countries in terms of the transfer protocol, including the timing of drug administration, and facility standards for ECPR. Hence, further investigations with prospective large international cohorts are required.

## Conclusions

We showed that patients with OHCA undergoing ECPR at HVCs have significantly higher survival rates than those undergoing the procedure at MVCs and LVCs, and that HVCs had a lower proportion of complications than LVCs.

### Supplementary Information

Supplementary Figure 1.

## Data Availability

The dataset supporting the conclusions of this study is available from the corresponding author on reasonable request. The data are not publicly available because of privacy and ethical restrictions.

## Abbreviations

AOR: Adjusted odds ratios
CI: Confidence interval
CPC: Cerebral performance categories
CPR: Cardiopulmonary resuscitation
CT: Computed tomography
ECMO: Extracorporeal membrane oxygenation
ECPR: Extracorporeal cardiopulmonary resuscitation
HVC: High-volume centers
LVC: Low-volume centers
MVC: Medium-volume centers
OHCA: Out-of-hospital cardiac arrest
PEA: Pulseless electrical activity
ROSC: Return of spontaneous circulation
