# Supplementary figures and images for: Impact of center volume on in-hospital mortality in adult patients with out‑of‑hospital cardiac arrest resuscitated using extracorporeal cardiopulmonary resuscitation: a secondary analysis of the SAVE-J II study
Source: Sci Rep. 2024 Apr 9;14:8309. doi: 10.1038/s41598-024-58808-y (PMC11003956; doi:10.1038/s41598-024-58808-y)

**Additional Figure 1.** Number of annual ECPR cases per year at each institution.

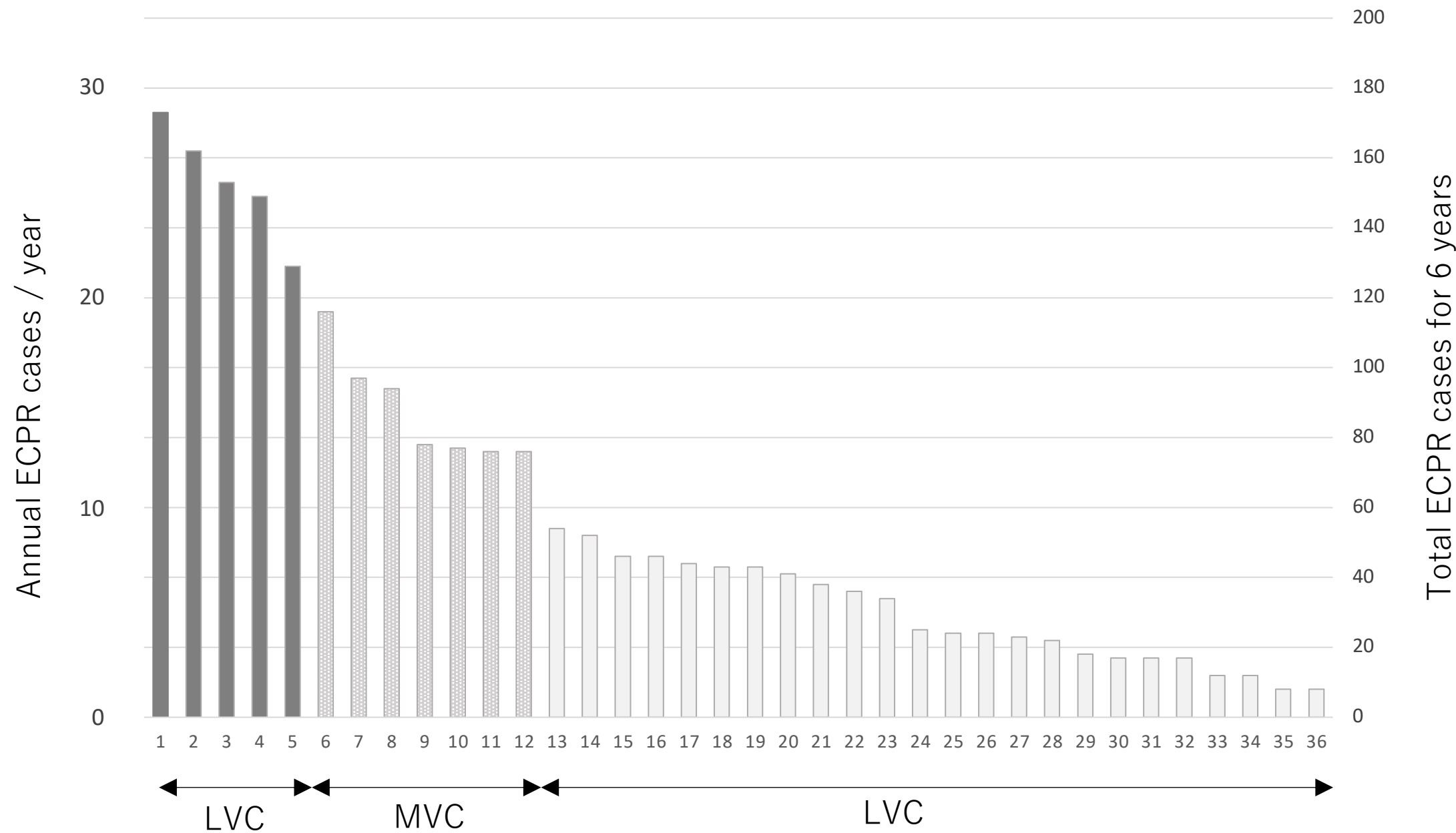

Supplement: Supplementary file 1 — Supplementary Figure 1. [file 41598_2024_58808_MOESM1_ESM.pdf]
